# Impact of GAS5 genetic polymorphism on prostate cancer susceptibility and clinicopathologic characteristics

**DOI:** 10.7150/ijms.38080

**Published:** 2019-09-20

**Authors:** Chia-Yen Lin, Shian-Shiang Wang, Cheng-Kuang Yang, Jian-Ri Li, Chuan-Shu Chen, Sheng-Chun Hung, Kun-Yuan Chiu, Chen-Li Cheng, Yen-Chuan Ou, Shun-Fa Yang

**Affiliations:** 1 Institute of Medicine, Chung Shan Medical University, Taichung, Taiwan; 2 Division of Urology, Department of Surgery, Taichung Veterans General Hospital, Taichung, Taiwan; 3 Division of Surgical Critical Care, Department of Critical Care Medicine, Taichung Veterans General Hospital, Taichung, Taiwan; 4 Department of Applied Chemistry, National Chi Nan University, Nantou, Taiwan; 5 Department of Medicine and Nursing, Hungkuang University, Taichung, Taiwan; 6 Department of Urology, Tung's Taichung MetroHarbor Hospital, Taichung, Taiwan; 7 Department of Medical Research, Chung Shan Medical University Hospital, Taichung, Taiwan

**Keywords:** GAS5, prostate cancer, polymorphism, prognosis

## Abstract

Down-regulation of Growth arrest-specific 5 (GAS5) is correlated with enhanced cell proliferation and poorer prognosis of prostate cancer. We aimed to investigate the effect of variant rs145204276 of GAS5 on the prostate cancer susceptibility and clinicopathologic characteristics. In this study, 579 prostate cancer patients who underwent robot-assisted radical prostatectomy and 579 healthy controls were included. The frequency of the allele del of rs145204276 were compared between the patients and the controls to evaluate the impact of tumor susceptibility and the correlation of clinicopathological variables. The results shown that patients who carries genotype ins/del or del/del at SNP rs145204276 showed decreased risk of pathological lymph node metastasis disease (OR=0.545, p=0.043) and risk of seminal vesicle invasion (OR=0.632, p=0.022) comparing to with genotype ins/ins. In the subgroup analysis of age, more significant risk reduction effects were noted over lymph node metastasis disease (OR=0.426, p=0.032) and lymphovascular invasion (OR=0.521, p=0.025). In conclusion, the rs145204276 polymorphic genotype of GAS5 can predict the risk of lymph node metastasis. This is the first study to report the correlation between GAS5 gene polymorphism and prostate cancer prognosis.

## Introduction

Prostate cancer (PCa) is one of the most prevalent malignancy in male gender at developed country. There are estimated 174650 newly diagnosed cases and 31620 cancer-related death in the United States alone in 2019 1. The incidence of PCa increases with advancing age. And 64% of new diagnosed cases were older than age 65 years 2. Consider the relative limited life expectancy; the older patients are more likely to receive active surveillance and observation, instead of potentially curative local therapy. However, the older patients were reported to get high-risk prostate cancer at diagnosis more frequently 3. And age is also known as a risk factor of pathological upgrading to higher risk disease after radical prostatectomy 4. The older men with high-risk disease, treated local therapy, had a 46 % reduction of mortality risk comparing with who treated conservatively 3. In current practice, the older PCa patients often received insufficient diagnostic survey and subsequent curative treatment 5. In order to balance between cancer specific mortality and overtreatment of these older patients, further prognostic factors to identify who needed aggressive cancer treatment is an important issue.

In the past, the screening of prostate cancer was based on elevated prostate specific antigen (PSA) and digital examination. And we classified risk of PCa progression combining with tumor stage, PSA and Gleason score of tumor grading. However, there are numerous newly published evidences indicated the importance of genetic features (both genomic alteration and single nucleotide polymorphism) in PCa prediction and prognosis 6-9.

Recently, studies about tumor biology started focusing not only the coding sequence but also evaluating the impact of long noncoding RNAs (LncRNAs). LncRNAs defined as longer than 200 nucleotides and do not have ability to translate 10. Although not in charge of protein coding, the LncRNAs can have regulatory effects of cell differentiation, migration, proliferation and apoptosis by interacting with DNA, RNA and proteins 11-13. And genetic variant over the promoter region of LncRNA was reported to modulate the expression level by methylation 14.

Growth arrest-specific 5 (GAS5), a LncRNA encoded by the GAS5 gene, is recently identified as a tumor suppressor in several cancers such as lung, breast, prostate and colorectal cancers 15. Although the exact expression level of GAS5 in PCa cell is still controversy, GAS5 is thought to play an important role in the proliferation, invasion, migration, and metastasis of PCa cells 16-18. The expression of GAS5 was identified to downregulate microRNA-21(miR-21)/miR-1284, then increase the expression PTEN/ PCDC4/AKT and result in cell apoptosis and limit proliferation of prostate cancer cell 19.

Single nucleotide polymorphism (SNP) is defined as a single nucleotide from the shared genome sequence changed more than 1% within a population 20. Several genetic polymorphisms had been associated with PCa risk, tumor grading and PCa specific mortality 21-23. The variant rs145204276, shown as “-/AGGCA “, is a 5-bp indel polymorphism in the GAS5 promoter region. Rs145204276 was reported to affect expression of GAS5 and increase susceptibility of several cancers 24-26. Moreover, this SNPs of GAS5 gene was reported significantly affecting the gleason score, disease stage and prognosis of prostate cancer 19. But, there are only 158 PCa patients included in this study, and this sample size is a little below power to conclude the susceptibility of prostate cancer and SNP of GAS5. Our study design is to further test the effect of SNP rs145204276 of GAS5 in PCa patient. To our knowledge, this study has the biggest sample size to test correlation of the SNP of GAS5 and cancer susceptibility and clinicopathologic characteristics of PCa patients in Taiwan to date.

## Materials and Methods

### Description of enrolled subjects

In this study, we enrolled 579 patients with adenocarcinoma of prostate, underwent robotic assisted radical prostatectomy from 2012 to 2017 at Taichung Veteran General Hospital. At the same time, 579 age-matched individuals were also included as healthy control. Before opening of this study, the approval was certified by the Institutional Review Broad (IRB) of Taichung Veteran General Hospital, and the informed consent was written by each participant (IRB No. CE19062A). The medical information for each patient was acquired from personal medical records, including initial PSA level at diagnosis, Gleason score of initial biopsy, clinical and pathological TNM staging, Gleason grade group 27, D'Amico classification 28 and all the pathological features of permanent pathological result.

### Specimen collection and Genomic DNA extraction

Whole blood samples were collected from controls and PCa patients. There were 579 PCa patients included and all the blood sample were obtained before surgery. The specimens were placed in tubes with EDTA, centrifuged immediately then stored at -80 ^o^C. Genomic DNA was extracted from whole blood sample with QIAamp DNA blood mini kits (Qiagen, Valencia, CA, USA) based on the manufacture's instruction as described previously 29. DNA was dissolved with TE buffer and stored at -20°C before Real-time PCR analysis.

### Selection of GAS5 genetic polymorphism

The GAS5 variant rs145204276 is a 5-bp indel polymorphism located at the promoter region. Rs145204276 was selected in this study since this SNP was associated with the progression of several cancers 24-26. The SNP rs145204276 of GAS5 was genotyped with TaqMan assay with SDS 3.0 software (Applied Biosystems, Foster City, CA, USA) and interpreted with StepOne™ Real-time PCR (RT-PCR) System (Applied Biosystems, Foster City, CA, USA).

### Statistical analysis

A goodness-of-fit v2 test was used to exam Hardy-Weinberg equilibrium for biallelic markers. Mann-Whitney U test were used to evaluate the differences among demographic characteristics between PCa group and controls. The odds ratios (ORs) with 95% confidence intervals (CIs) were calculated using logistic regression models to estimate the association between genotypic frequencies and different clinicopathological characteristics. The statistical significant difference was defined as p<0.05. All the data were analyzed using Statistical Analytic System (SAS Institute, Cary, NC, USA) software (vers. 9.1, 2005) for Windows.

## Results

### Characteristics of Study Participants

The demographic characteristics in 579 patients were presented in this study (Table 1). At diagnosis, 334 patients (57.7%) were older than 65-year-old, 270 patients (46.6%) had initial PSA level more than 10 ng/mL, 501 patients (86.5%) were clinically localized disease (cT1+cT2). 273 and 49 patients had pathological proof locally advanced disease (pT3+pT4) (47.2%) and lymph node metastasis (8.5%) respectively. The percentages of low-, intermediate-, and high-risk PCa according to D'Amico classification were 10.4% (60), 38.0% (220), and 51.6% (299).

### Association of GAS5 gene polymorphisms and cancer susceptibility and clinical status of PCa

The allele frequency of GAS5 rs145204276 SNP in the patients with PCa and non-cancer controls is shown in Table 2. In our recruited control group, the frequencies of GAS5 rs145204276 (χ^2^ value: 0.132, p=0.717) was in Hardy-Weinberg equilibrium. However, there are no significant correlations noted in all codominant/dominant/recessive/additive models. In the table 3, we evaluate the association of clinicopathologic characteristics of patients with PCa and GAS5 rs145204276 polymorphism. Patients who carries genotype ins/del or del/del showed decreased risk of pathological lymph node metastasis disease (OR=0.545; 95% CI=0.301-0.988, p=0.043) and risk of seminal vesicle invasion (OR=0.632; 95% CI=0.426-0.939, p=0.022) comparing to with genotype ins/ins. The risk of lymphovascular invasion is also slightly lower in patients who carry at least one del phenotype but there is no statistical significance (OR=0.647; 95% CI=0.435-1.044, p=0.076). And no difference was noted in other well-known prognostic factors such as initial PSA level, pathological gleason grade group, clinical/pathological T stage and D'Amico risk classification.

### Correlation of GAS5 SNPs and clinical status of PCa with age over 65 years

In the table 4, we evaluate the association of GAS5 rs145204276 polymorphism and clinicopathologic characteristics of patients with PCa and older than 65 years. Patients who carries GAS5 rs145204276 ins/del or del/del showed decreased risk of clinical locally advanced disease (OR=0.513; 95% CI=0.286-0.923, p=0.024), pathological lymph node metastasis disease (OR=0.462; 95% CI=0.225-0.946, p=0.032) and lymphovascular invasion (OR=0.521; 95% CI=0.292-0.927, p=0.025) comparing to with genotype ins/ins. The risk of higher initial PSA level (> 10 ng/mL) and seminal vesicle invasion are also slightly lower in patients who carry at least one del phenotype but there is no statistical significance (OR=0.676; 95% CI=0.435-1.049, p=0.080; and OR=0.613; 95% CI=0.370-1.018, p=0.057, respectively). And no difference was noted in other well-known prognostic factors such as pathological gleason grade group, pathological T stage and D'Amico risk classification.

## Discussion

Recently, increasing evidence indicated that decreasing expression of GAS5 can affect the susceptibility of many kinds of cancers and associated with poorer prognosis of hepatocellular carcinoma, cervical cancer, renal cancer, lung cancer, gastric cancer and melanoma 24-26, 30-32. The rs145204276 ins/del polymorphism can regulate the expression of GAS5 through affecting one CpG island methylation condition 24. Cancer patients with allele del of rs145204276 were also found to have remarkable higher expression of GAS5 in several different cancer tissues, and prostate cancer is one of them 19, 24-26, 33. The del allele of rs145204276 was also significantly correlated with decrease risk of lung cancer, hepatocellular carcinoma and gastric cancer 24-26. However, in our study, the del allele of rs145204276 did not affect the tumor susceptibility of prostate cancer. The result did not change when evaluating with codominant, dominant, recessive or additive model. The reason might be the risk of prostate cancer had already connected with numerous SNPs 34, 35. Thus, the effect of GAS5 SNP about prostate cancer susceptibility became attenuated. To the best of our knowledge, this is the first study to evaluate the genetic polymorphism of GAS5 with susceptibility of prostate cancer.

In the analysis about clinicopathologic characteristics of prostate cancer in whole population, SNP rs145204276 was significantly associated with decreasing risk of pathological proved lymph node metastasis (OR=0.545; 95% CI=0.301-0.988, p=0.043) and risk of seminal vesicle invasion (OR=0.632; 95% CI=0.426-0.939, p=0.022). There was also a better trend of decreasing lymphovascular invasion (OR=0.647; 95% CI=0.435-1.044, p=0.076). Lymphovascular invasion, also known as minimal lymphatic involvement, has been frequently reported as a prognostic factor to predict biochemical recurrence (BCR) after radiotherapy and radical prostatectomy 36-39. In the cause analysis of BCR, the increasing risk was mainly from progression to lymph node metastasis instead of residual tumor cells of positive surgical margins 39. A recent study reported that the lymphovascular invasion of PCa cells has a significantly prognostic impact on disease progression.^40^

Although we cannot provide the direct impact of SNP rs145204276 and the survival outcome, but considering the presence of lymph node metastasis after radical prostatectomy is a well-known poor prognostic factor with increased long-term risk of cancer specific mortality, estimated to range from 20% to 42% 41-43. A worse cancer specific survival in patient with SNP rs145204276 might be a reasonable expectation, but further direct evidence to validate is still needed.

In the subgroup analysis of PCa patients with age classification, we realized the risk reduction effect of lymph node metastasis in patients with SNP rs145204276 is mainly contributed by the elders. PCa patients with age more than 65 year and carrying SNP rs145204276 are correlated to a 57.4% risk reduction of pathological proved lymph node metastasis (OR=0.426; 95% CI=0.225-0.946, p=0.032) and 47.9% risk reduction of lymphovascular invasion (OR=0.521; 95% CI=0.292-0.927, p=0.025) significantly. And these risk reduction effects were not revealed in the age below or equal to 65 years' population.

In the current real world practice of PCa treatment, the older patients are more likely to receive active surveillance and observation, instead of potentially curative local therapy because of the relative limited life expectancy. However, the older patients were reported to have more aggressive prostate cancer at diagnosis more frequently 3. And age is also considered as a risk factor of pathological upgrading to higher risk disease after operation^4^. In older men with high-risk disease, the aggressive treatment with a 46 % reduction of mortality risk was reported comparing to treat conservatively 3. To balance between survival benefit and overtreatment of these older patients, further prognostic factors to initiate or avoid aggressive cancer treatment is an important issue. And GAS5 SNP rs145204276 could be part of the resolution. In our study, GAS5 SNP rs145204276 showed a strong prevention effect of lymphatic spreading in PCa patient older than 65 years. In the future study, the precise genetic mechanism with multifactorial evaluation of prostate cancer pathogenesis and prognosis is worthy for investigation.

In conclusion, although the GAS5 SNP rs145204276 did not affect the PCa susceptibility, our study indicated men with PCa and carrying GAS5 SNP rs145204276 are less likely to develop lymph node metastasis, especially in the age older than 65 years' group.

## Figures and Tables

**Table 1:** The distributions of demographical characteristics in 579 patients with prostate cancer.

Variable	Patients (N=579)
**Age at diagnosis (years)**	
≤ 65	245 (42.3 %)
> 65	334 (57.7 %)
**PSA at diagnosis (ng/mL)**	
≤ 10	309 (53.4 %)
> 10	270 (46.6 %)
**Pathologic Gleason grade group**	
1+2+3	484 (83.6 %)
4+5	95 (16.4 %)
**Clinical T stage**	
1+2	501 (86.5 %)
3+4	78 (13.5 %)
**Pathologic T stage**	
2	306 (52.8 %)
3+4	273 (47.2 %)
**Pathologic N stage**	
N0	530 (91.5 %)
N1	49 (8.5 %)
**Seminal vesicle invasion**	
No	452 (78.1 %)
Yes	127 (21.9 %)
**Perineural invasion**	
No	155 (26.8 %)
Yes	424 (73.2 %)
**Lymphovascular invasion**	
No	482 (83.2 %)
Yes	97 (16.8 %)
**D'Amico classification**	
Low risk	60 (10.4 %)
Intermediate risk	220 (38.0 %)
High risk	299 (51.6 %)

**Table 2:** Associations between GAS5 rs145204276 and 579 patients with prostate cancer.

Genetic model	Genotype	Controls (N=579) n (%)	Patients (N=579) n (%)	OR (95% CI)	p value
**Codominant model**	Ins/Ins	237 (40.9%)	263 (45.4%)	1.000	
	Ins/Del	270 (46.7%)	252 (43.5%)	0.841 (0.658-1.075)	p=0.167
	Del/Del	72 (12.4%)	64 (11.1%)	0.801 (0.548-1.171)	p=0.252
**Dominant model**	Ins/Ins	237 (40.9%)	263 (45.4%)	1.000	
	Ins/Del + Del/Del	342 (59.1%)	316 (54.6%)	0.833 (0.660-1.051)	p=0.123
**Recessive model**	Ins/Ins + Ins/Del	507 (87.6%)	515 (88.9%)	1.000	
	Del/Del	72 (12.4%)	64 (11.1%)	0.875 (0.612-1.252)	p=0.465
**Additive model**	Ins allele	744 (64.2%)	778 (67.2%)	1.000	
	Del allele	414 (35.8%)	380 (32.8%)	0.878 (0.739-1.042)	p=0.137

The odds ratios (ORs) and with their 95% confidence intervals (CIs) were estimated by logistic regression models.

**Table 3:** Odds ratio (OR) and 95% confidence interval (CI) of clinical status and *GAS5* rs145204276 genotypic frequencies in 579 patients with prostate cancer.

Variable	Genotypic frequencies			
rs145204276	ins/ins (N=263)	ins/del + del/del (N=316)	OR (95% CI)	p value
**PSA at diagnosis (ng/mL)**				
≤ 10	148 (56.3%)	161 (50.9%)	1.00	p=0.201
> 10	115 (43.7%)	155 (49.1%)	1.239 (0.892-1.721)	
**Pathologic Gleason grade group**				
1+2+3	216 (82.1%)	268 (84.8%)	1.00	p=0.386
4+5	47 (17.9%)	48 (15.2%)	0.823 (0.530-1.278)	
**Clinical T stage**				
1+2	223 (84.8%)	278 (88.0%)	1.00	p=0.264
3+4	40 (15.2%)	38 (12.0%)	0.762 (0.473-1.229)	
**Pathologic T stage**				
2	140 (53.2%)	166 (52.5%)	1.00	p=0.867
3+4	123 (46.8%)	150 (47.5%)	1.029 (0.741-1.427)	
**Pathologic N stage**				
N0	234 (89.0%)	296 (93.7%)	1.00	p=0.043*
N1	29 (11.0%)	20 (6.3%)	0.545 (0.301-0.988)	
**Seminal vesicle invasion**				
No	194 (73.8%)	258 (81.6%)	1.00	p=0.022*
Yes	69 (26.2%)	58 (18.4%)	0.632 (0.426-0.939)	
**Perineural invasion**				
No	63 (24.0%)	92 (29.1%)	1.00	p=0.163
Yes	200 (76.0%)	224 (70.9%)	0.767 (0.528-1.114)	
**Lymphovascular invasion**				
No	211 (80.2%)	271 (85.8%)	1.00	p=0.076
Yes	52 (19.8%)	45 (14.2%)	0.674 (0.435-1.044)	
**D'Amico classification**				
Low risk/ Intermediate risk	129 (49.0%)	151 (47.8%)	1.00	p=0.762
High risk	134 (51.0%)	165 (52.2%)	1.052 (0.758-1.459)	

The ORs with analyzed by their 95% CIs were estimated by logistic regression models. * p value < 0.05 as statistically significant.

**Table 4:** Odds ratio (OR) and 95% confidence interval (CI) of clinical status and *GAS5* rs145204276 genotypic frequencies in 334 prostate cancer patients with age over 65 years.

Variable	Genotypic frequencies			
rs145204276	ins/ins (N=157)	ins/del + del/del (N=177)	OR (95% CI)	p value
**PSA at diagnosis (ng/mL)**				
≤ 10	57 (36.3%)	81 (45.8%)	1.00	p=0.080
> 10	100 (63.7%)	96 (54.2%)	0.676 (0.435-1.049)	
**Pathologic Gleason grade group**				
1+2+3	122 (77.7%)	146 (82.5%)	1.00	p=0.274
4+5	35 (22.3%)	31 (17.5%)	0.740 (0.431-1.270)	
**Clinical T stage**				
1+2	123 (78.3%)	155 (87.6%)	1.00	p=0.024*
3+4	34 (21.7%)	22 (12.4%)	0.513 (0.286-0.923)	
**Pathologic T stage**				
2	78 (49.7%)	91 (51.4%)	1.00	p=0.752
3+4	79 (50.3%)	86 (48.6%)	0.933 (0.607-1.434)	
**Pathologic N stage**				
N0	134 (85.4%)	164 (92.7%)	1.00	p=0.032*
N1	23 (14.6%)	13 (7.3%)	0.462 (0.225-0.946)	
**Seminal vesicle invasion**				
No	112 (71.3%)	142 (80.2%)	1.00	p=0.057
Yes	45 (28.7%)	35 (19.8%)	0.613 (0.370-1.018)	
**Perineural invasion**				
No	33 (21.0%)	50 (28.2%)	1.00	p=0.127
Yes	124 (79.0%)	127 (71.8%)	0.676 (0.408-1.119)	
**Lymphovascular invasion**				
No	122 (77.7%)	154 (87.0%)	1.00	p=0.025*
Yes	35 (22.3%)	23 (13.0%)	0.521 (0.292-0.927)	
**D'Amico classification**				
Low risk/ Intermediate risk	62 (39.5%)	77 (43.5%)	1.00	p=0.458
High risk	95 (60.5%)	100 (56.5%)	0.848 (0.548-1.312)	

The ORs with analyzed by their 95% CIs were estimated by logistic regression models. * p value < 0.05 as statistically significant.
